# Oriented Soft DNA Curtains for Single-Molecule Imaging

**DOI:** 10.1021/acs.langmuir.1c00066

**Published:** 2021-03-09

**Authors:** Aurimas Kopu̅stas, Šaru̅nė Ivanovaitė, Tomas Rakickas, Ernesta Pocevičiu̅tė, Justė Paksaitė, Tautvydas Karvelis, Mindaugas Zaremba, Elena Manakova, Marijonas Tutkus

**Affiliations:** ^†^Departments of Molecular Compound Physics, ^‡^Nanoengineering, and ^§^Functional Materials and Electronics, Center for Physical Sciences and Technology, Savanoriu 231, Vilnius LT-02300, Lithuania; ∥ Life Sciences Center, Institute of Biotechnology, Vilnius University, Saulėtekio av. 7, LT-10257 Vilnius, Lithuania

## Abstract

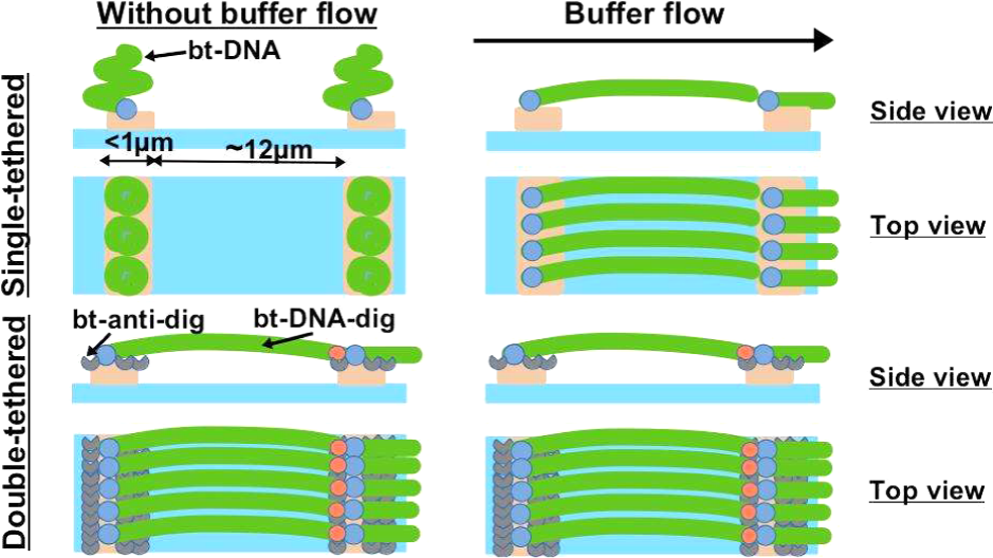

Over the past 20 years, single-molecule methods have become extremely important for biophysical studies. These methods, in combination with new nanotechnological platforms, can significantly facilitate experimental design and enable faster data acquisition. A nanotechnological platform, which utilizes a flow-stretch of immobilized DNA molecules, called DNA Curtains, is one of the best examples of such combinations. Here, we employed new strategies to fabricate a flow-stretch assay of stably immobilized and oriented DNA molecules using a protein template-directed assembly. In our assay, a protein template patterned on a glass coverslip served for directional assembly of biotinylated DNA molecules. In these arrays, DNA molecules were oriented to one another and maintained extended by either single- or both-end immobilization to the protein templates. For oriented both-end DNA immobilization, we employed heterologous DNA labeling and protein template coverage with the antidigoxigenin antibody. In contrast to single-end immobilization, both-end immobilization does not require constant buffer flow for keeping DNAs in an extended configuration, allowing us to study protein–DNA interactions at more controllable reaction conditions. Additionally, we increased the immobilization stability of the biotinylated DNA molecules using protein templates fabricated from traptavidin. Finally, we demonstrated that double-tethered Soft DNA Curtains can be used in nucleic acid-interacting protein (e.g., CRISPR-Cas9) binding assay that monitors the binding location and position of individual fluorescently labeled proteins on DNA.

## Introduction

Dynamic protein–nucleic acid (NA) interactions play a crucial role in the regulation of many cellular processes. Currently, these problems are widely investigated using advanced microscopy-based methods that enable direct monitoring of NA–protein interactions at the single-molecule (SM) level in real time. Information obtained from these experiments is crucially important for building mechanistic models of diverse reactions.^1,2^ Nano- or microscopic platforms combined with microscopy techniques become very popular and allow accessing information that is otherwise hidden.^3−5^ However, most of the SM techniques cannot be parallelized and are often technically challenging. Therefore, new high-throughput platforms for SM imaging of protein–NA interactions are in high demand.^6^

One of the best combinations of SM methods with a nanotechnological platform was the development of the deoxyribonucleic acid (DNA) Curtains platform. It enabled high-throughput SM imaging by employing nanoengineering, microfluidics, supporting lipid bilayers (SLBs), and SM microscopy.^6−8^ This platform utilizes an inert lipid bilayer, which passivates the otherwise sticky surface of the flowcell channel, and mechanical barriers to partition the lipids. Biotinylated DNA molecules that are anchored on the biotinylated lipids via neutravidin (nAv) can be manipulated using hydrodynamic force. Another similar recently developed platform is called DNA skybridge.^9^ It utilizes a structured poly(dimethylsiloxane) (PDMS) surface for DNA immobilization and a thin Gaussian light sheet beam parallel to the immobilized DNA for visualization of DNA and protein interaction at the SM level. Also, there are several other prior approaches that array DNA on passivated surfaces for DNA–protein interaction studies.^10−13^ The original DNA Curtains platform demonstrated great benefits for studies of many different NA-interacting proteins. However, the original DNA Curtains are less stable and more expensive fabrication-wise than the platform described in this and our previous work.^14^ The skybridge platform contains stably immobilized DNA molecules, but it utilizes rather unusual phenomena for visualization of fluorescently labeled DNA and proteins. However, it is an interesting alternative to the existing DNA Curtains platform.

Recently, we demonstrated that streptavidin (sAv) patterns on the modified coverslip surface can be utilized to fabricate biotinylated DNA arrays.^14^ The design of the protein patterns on the modified surface ensures predefined distribution and aligning of the biotinylated DNA molecules on the narrow line-features (>200 nm). We refer to these aligned molecules as Soft DNA Curtains. The application of hydrodynamic buffer flow allows extension of the immobilized DNA molecules along the surface of the flowcell channel. These Soft DNA Curtains permit simultaneous visualization of hundreds of individual DNA molecules that are aligned with respect to one another and offer parallel data acquisition of diverse biological systems. We showed that Soft DNA Curtains are easy to fabricate in any laboratory having access to an atomic force microscope (AFM) and objective or prism-based total internal reflection fluorescence microscopy (TIRF).

One of the drawbacks of our previous work was that the double-tethered Soft DNA Curtains had no defined orientation of both-end biotinylated DNA molecules. Such DNA molecules could bind to the sAv line-feature in any direction. Random orientation of DNAs would not create a huge problem because one end of the DNA molecule could be fluorescently labeled and this labeling would allow us to postorient DNA molecules during data analysis. However, this procedure introduces an extra complication of the experiment.

Here, we fabricated the uniformly oriented double-tethered DNA Curtains using heterologous labeling of the DNA molecules by biotin and digoxigenin (dig). We confirmed the defined orientation of DNA molecules using a fluorescent tag introduced asymmetrically to the DNA molecule. The well-controlled fabrication procedure of high-quality protein templates was achieved using a portable printing device (PPD) developed especially for this purpose. We increased the stability of the immobilized DNA molecules using a more stable alternative to sAv called traptavidin (tAv)^15,16^ as an ink for the fabrication of protein templates. These improvements allowed us to demonstrate that double-tethered Soft DNA Curtains can be used in the NA-interacting protein binding assay that monitors the binding location and position of fluorescently labeled CRISPR-Cas9 proteins on DNA.

## Materials and Methods

### Chemicals and Materials

Silicone elastomer Sylgard 184 (Dow, Midland, MI) was used for lift-off microcontact printing (μCP) stamp production. For Si master structure production, gold-coated silicon wafers (a 20 nm-thick Au film and a 2 nm Ti adhesion layer, Ssens BV, The Netherlands) were used. Before use, substrates were cleaned in SC-1 solution: ultrapure water, 30% hydrogen peroxide (Carl Roth GmbH, Germany), 25% ammonia solution (Carl Roth GmbH, Germany) at 5:1:1 v/v/v. Wet chemical etching solution for Au was as follows: 20 mM Fe(NO_3_)_3_·9H_2_O (Fluka, Switzerland), 30 mM thiourea (Fluka, Switzerland), and 1 mM HCl (Sigma-Aldrich) dissolved in ultrapure water saturated with octanol (Sigma-Aldrich). DNA primers were synthesized and purified by Iba-lifesciences (Germany) or Metabion (Germany). The other materials that were used were as follows: nitrogen gas (purity of 99.999%, ElmeMesser Lit, Lithuania), ultrapure water (Synergy 185 UV, Millipore or Labostar, Siemens), ethanol (99.9%, Merck KGaA, Germany), streptavidin (SERVA, Germany), *N*-(2-hydroxyethyl)piperazine-*N*′-ethanesulfonic acid (HEPES; Carl Roth GmbH, Germany), Tris-acetate (Sigma-Aldrich), NaCl (Carl Roth GmbH, Germany), biotin-PEG4-NHS (Jena Bioscience, Germany), and Anti-Dig antibodies (Roche). Buffer solutions were as follows: (A) 33 mM Tris-acetate (pH = 7.9, at 25 °C), 66 mM K-Acetate; (B) 40 mM Tris (pH = 7.8, at 25 °C); and (C) 20 mM HEPES (pH = 7.5, at 25 °C), 150 mM NaCl.

### Production and Purification of Proteins

His-tagged tAv was produced and purified according to the published protocol.^15,16^*Escherichia coli* BL21(DE3) cells were transformed with the pET21a tAv plasmid, plated onto Luria-Broth (LB)-Carbenicillin agar plates, and incubated at 37 °C overnight. An overnight culture in LB-Ampicillin was grown out of a single colony with shaking at 220 r.p.m. and 37 °C. The overnight culture was diluted 100-fold into an LB-Ampicillin medium, grown at 37 °C until OD_600_ 0.9, and the protein expression was induced with 0.5 mM isopropyl-β-d-thiogalactopyranoside for 4 h at 37 °C. Cells were collected by centrifugation at 5000*g* and 4 °C for 10 min. The cell pellet was resuspended in a lysis buffer (300 mM NaCl, 50 mM Tris, 5 mM ethylenediaminetetraacetic acid (EDTA), 0.8 mg/mL lysozyme, 1% Triton X-100 (pH = 7.8, at 25 °C)) and put on a rocker at 80 r.p.m. at room temperature for 20 min. Pulsed sonication of the cell pellet on ice at 30% amplitude was performed afterward for 10 min. Centrifugation at 27 000*g* and 4 °C for 15 min followed by washing of the inclusion body pellet in a wash buffer (100 mM NaCl, 50 mM Tris, 0.5% Triton X-100 (pH = 7.8, at 25 °C)) was repeated three times. Isolated inclusion bodies were dissolved in 6 M guanidinium hydrochloride (pH = 1.5, at 25 °C) and then spun at 17 700*g* and 4 °C for 20 min. Protein precipitation using solid ammonium sulfate was then carried out to precipitate tAv from their refolds. The precipitate was resuspended in a minimal volume of phosphate-buffered saline (PBS) at room temperature, centrifuged at 14 000*g* and 4 °C for 5 min, and the excess of ammonium sulfate was removed by running the supernatant through a NAP-25 column (GE Healthcare). The tAv was purified using a HiTrap chelating column (GE Healthcare) charged with Ni^2+^ equilibrated with an equilibration buffer (300 mM NaCl, 50 mM Tris-hydrochloride (pH = 7.8, at 25 °C)). Protein was eluted with an elution buffer (300 mM NaCl, 50 mM Tris, 0.5 M imidazole (pH = 7.8, at 25 °C)). The fractions containing tAv were dialyzed into PBS at 4 °C and concentrated by ultrafiltration using a 9 kDa MWCO centrifugal concentrator. The final yield of purification was 3 mg of tAv per 1 L of initial culture. Wild-type *Streptococcus pyogenes* (Sp) Cas9 was expressed and purified as published previously.^17^

### Production of DNA

Biotinylated oligonucleotides were annealed to the overhang (cos sequences) at either the left or both ends of bacteriophage λ DNA (48.5 kb, ThermoFisher Scientific). The sequences of the oligonucleotides were as follows: 5′-AGGTCGCCGCCC[TEG-digoxigenin]-3′ (right end) and 5′-GGGCGGCGACCT-TEG[Biotin]-3′ (left end) (Metabion). These two oligonucleotides were phosphorylated at the 5′-end using a polynucleotide kinase (PNK, ThermoFisher Scientific) reaction (1 μM of the respective oligonucleotide, 10× diluted PNK, PNK buffer, 0.1 mM adenosine triphosphate, ATP) at 37 °C for 30 min. PNK was inactivated by incubation for 5 min at 95 °C. The λ DNA and the oligonucleotide were mixed at the molar ratio of 1:10, heated to 80 °C, and slowly cooled to room temperature. Subsequently, T4 DNA ligase (ThermoFisher Scientific) was added, and the reaction mixture was incubated at room temperature for 2 h. After the reaction was complete, the DNA ligase was inactivated by heating to 70 °C for 10 min, the excess oligonucleotide was removed using a CHROMA SPIN TE-1000 column (Clontech), and the purified DNA was stored at −20 °C.

For the insertion of an ATTO647N-labeled oligonucleotide complementary to the position 14 711 bp from the biotinylated end of the λ DNA, we employed the previously described strategy^18^ and followed the more recently described procedure.^19^ First, 2 μg of λ DNA was incubated for 2 h with the nicking enzyme Nt.BstNBI (20 units, NEB) at 50 °C in the nickase buffer. The nicked DNA was mixed with a 100-fold excess of three oligonucleotides: (5′-pTTCAGAGTCTGACTTTT[ATTO647N]-3′), (5′-AGGTCGCCGCCC[TEG-digoxigenin]-3′), and (5′-GGGCGGCGACCT-TEG[Biotin]-3′). The mixture was incubated at 55 °C for 20 min and then cooled down at a rate of 0.5 °C/min to 16 °C prior to ligation. Next, ATP was added to a final concentration of 1 mM along with 50 units of T4 ligase (ThermoFisher Scientific). The ligation reaction mixture was incubated at room temperature overnight. Any remaining nicking or ligase activity was quenched by adding 20 mM EDTA. The excess oligonucleotides were removed using a CHROMA SPIN TE-1000 column (Clontech), and the purified DNA was stored at −20 °C.

Biotinylated 5 kb-long DNA was synthesized by polymerase chain reaction (PCR) using ΦX174 RF1 DNA (ThermoFisher Scientific) as a template and oligonucleotides 5′ biotin-CGAAGTGGACTGCTGGCGG-3′ and 5′-CGTAAACAAGCAGTAGTAATTCCTGCTTTATCAAG-3′ as primers. The product was purified using the GeneJET PCR purification kit (ThermoFisher Scientific).

### Fabrication and Characterization of a Silicon Master

Si masters were fabricated and characterized according to the previously published procedure.^14^ Fabrication of the Si master involves formation of a self-assembled monolayer from 1-eicosanethiol,^20^ surface patterning by the nanoshaving lithography technique using an AFM (NanoWizard3, JPK Instruments AG, Germany), and wet chemical etching.^21−23^ Characterization of the Si master was performed using an upright optical microscope BX51 (Olympus, Japan) and the atomic force microscopy (AFM), operating in the AC mode.

### Characterization of Printed Protein Features

The width and morphology of the printed protein features on the glass surface were analyzed with an AFM in buffer C. For that, the glass sample was mounted into the ECCel (JPK Instruments AG, Germany) and imaged using the QI-Advanced mode. Before each measurement, the probe sensitivity and spring constant were calibrated using the contact-free calibration routine (based on the thermal spectrum of cantilever oscillations) built into AFM software. The setpoint for measurements was set to 1.5–2 nN tip pushing force.

### Protein Nanopatterning by Lift-Off μCP and the Portable Printing Device

Flat PDMS elastomer stamps for protein lift-off μCP were fabricated according to the previously published procedure.^14^ Briefly, the prepolymer and curing agent (10:1 ratio w/w, Sylgard 184 kit) were thoroughly mixed, degassed in a vacuum desiccator (30 min), poured into a plastic Petri dish, and cured in an oven (65 °C for 14 h). The thickness of the cast PDMS elastomer was ∼2 mm. The PDMS surface that was in contact with the Petri dish was treated as a flat one.

The lift-off μCP was performed similar to that in the published procedure.^14,24^ The PDMS elastomer (5 × 5 mm^2^ dimensions) was immersed in isopropanol for 10 min, held by tweezers, and dried for 15 s using N_2_ gas, and then placed on a plastic Petri dish and dried for another 10 min. The Si master was immersed in isopropanol (20 min), held by tweezers and dried, and cleaned by air plasma (5 min, ∼500 mTorr, high-power mode, PDC-002, Harrick). To homogeneously cover the PDMS surface with a film of the protein ink, the PDMS stamp was placed in a clean plastic Petri dish with its flat side facing up. Then, a 60 μL drop of a specified protein solution (in buffer A) was placed on the PDMS surface, mixed with the tip of a pipette, and kept for 10 min. After incubation, the protein ink was removed from the PDMS stamp by sucking it out with the pipette tip. Then, the PDMS stamp was held with tweezers and washed with 5 mL of buffer A using a 1 mL pipette, ∼50 mL of ultrapure water using a wash bottle, and dried under a N_2_ gas stream.

For the printing procedure, we build a PPD, which allowed us to apply different printing pressures (PPs; see the SI file for the detailed description). First, the cleaned Si master was placed on the silicon rubber at the bottom of the printing machine (Figure S2A). The dried PDMS stamp was placed facing the flat side-up on a piece of glass (10 × 10 mm^2^ dimensions), which was covered with a double-sided sticky tape (Figure S2B). Next, the PDMS stamp on the piece of glass was placed on the Si master using tweezers. The syringe holder was mounted on the top of the device, and pressure (PP = 0.6 mL, unless stated otherwise) was applied to the PDMS stamp on the Si master using the distant syringe (Figure S2C). The contact in between the patterned Si surface and the PDMS stamp was established for ∼15 s; then, the distant syringe was released and the glass slide with the PDMS elastomer was removed from the Si master using tweezers. Subsequently, the silanized and PEGylated (methoxy-poly(ethylene glycol) (PEG)-SVA and biotin-PEG-SVA, both 5 kDa, Lyasan Bio) glass coverslip (25 × 25 mm^2^, no. 1.5, Menzel Glaser) was placed instead of the Si master using tweezers. The PDMS stamp was transferred onto the PEGylated glass coverslip and kept for 1 min under pressure (PP = 0.6 mL, unless stated otherwise) applied by the distant syringe.

The surface of the glass coverslips was modified in the same way as described previously,^25^ using the biotin-PEG/methoxy-PEG (bt-PEG/m-PEG) ratio of 1:10 (w/w). To minimize nonspecific protein adsorption to the surface, we performed a second round of PEGylation with the short NHS-ester PEG molecules (333 Da) according to the published procedure.^26^

Next, the glass slide with the PDMS stamp was removed from the glass coverslip using tweezers and discarded. The patterned glass coverslip was assembled into the flowcell, which was prepared as described earlier.^14^ The Si masters were reused for the lift-off μCP multiple times, and in between the experiments, they were stored in 100% isopropanol. Storing in isopropanol helps remove PDMS residues.^27^

### TIRF Microscopy

The employed home-build TIRF microscopy setup was described previously.^14^ This microscopy setup was equipped with three different wavelength lasers: 488, 532, and 635 nm (all 20 mW, Crystalaser). These combined beams were directed to the objective (100×, 1.4NA, Nikon) using a quad-line dichroic mirror (zt405/488/532/640rpc, Chroma Technology Corp), which was placed in the upper filter cube turret installed in the microscope body (Nikon Eclipse Ti-U). The laser power before the objective was set to 2.5 mW for both 532 and 632 nm lasers and to 0.1 mW for the 488 nm laser. The exposure time of the EMCCD camera (Ixon3, Andor) was set to 100 ms. The microscopy images represented in the article and in the SI file were averaged over 10 consecutive frames, thus improving the signal-to-noise ratio. The penetration depth of the evanescent field was set to ∼300 nm for all wavelengths of excitation. This setup was equipped with a custom-built feed-back control system to compensate the *Z*-axis drift of the sample and keep it stably in focus. The average line quality factor was calculated using the formula described in our previous publication.^14^

### DNA Immobilization

First, the channel of the flowcell was filled with buffer B. To enhance surface passivation against nonspecific protein adsorption, we injected 5% Tween-20 solution in buffer B into the channel of the flowcell, incubated for 10 min, and washed out with 600 μL of buffer A.^28^ Next, the biotinylated DNA (∼30 pM, in buffer B) was added and incubated for at least 15 min. The excess of unbound DNA was washed out with ∼300 μL of buffer A. Then, DNA was labeled with the DNA intercalating green fluorescent dye, SYTOX green (SG, ThermoFisher Scientific), at a concentration of ∼0.4 nM (in imaging buffer: buffer A supplemented with 0.2% Tween-20, 1 mM dithiothreitol, DTT). The SG dye was present during the entire time of the experiment. In the case of the second DNA end tethering, the close-loop circulation was employed and 5 μL of biotin-anti-dig (bt-anti-dig) antibody was added (this resulted in ∼0.05 mg/mL concentration) and incubated for at least 10 min at low speed (∼0.1 mL/min). After 10 min, the speed of the buffer flow was increased to ∼1 mL/min and kept constant for 20 min. Then, to remove the excess of unbound bt-anti-dig, the flowcell was washed with 500 μL of buffer A in the open-loop circulation. Finally, 100 μL of imaging buffer was injected into the flowcell to reveal bound DNA.

### Cas9 Labeling and Complex Assembly

The SpCas9 complex was labeled using the ATTO647N conjugated oligonucleotide, which was hybridized with the tracrRNA 5′-end in the crRNA:tracrRNA duplex. Briefly, the tracrRNA 3′-modified for hybridization with the ATTO647N-labeled oligonucleotide was obtained using PCR with 5′-TAATACGACTCACTATAGGGCAAAACAGCATAGCAAGTTAAAATAAGG-3′ and 5′-GCGCACGAGCAAAAAGCACCGACTCGGTGCC-3′ primers from the pUC18 plasmid containing the RNA encoding sequence followed by in vitro transcription (TranscriptAid T7 high-yield transcription kit, ThermoFisher Scientific) and purification (GeneJET RNA purification kit, ThermoFisher Scientific). Next, the oligonucleotide 5′-TTGCGCACGAGCAAA-3′ (Metabion International AG), which is complementary to the tracrRNA 5′-end, was labeled with ATTO647N-NHS (1:60 DNA/dye molar ratio) and purified using a G-25 microspin column (Illustra, GE Healthcare). The measured labeling efficiency of the ATTO647N-oligonucleotide was >80%. Subsequently, the assembly of the crRNA:tracrRNA duplex and the hybridization of tracrRNA with the ATTO647N-oligonucleotide were performed simultaneously by mixing equimolar amounts of synthetic crRNA (Synthego), which contains the 5′-GAAATCCACTGAAAGCACAG-3′ target site, tracrRNA, and the ATTO647N-oligonucleotide along with 5× annealing buffer (Synthego), and then heating the mixture to 80 °C and allowing it to slowly cool down to room temperature. Finally, the Cas9–RNA complex was assembled from SpCas9 and crRNA/tracrRNA-ATTO647N (1:2 protein/RNA molar ratio) in a reaction buffer (10 mM Tris-HCl, pH = 7.5, at 37 °C, 100 mM NaCl, 1 mM EDTA, 0.5 mg/mL bovine serum albumin (BSA), 1 mM DTT) at 50 nM final concentration at 37 °C for 30 min. The complex was diluted to the concentration of 0.2 nM in the imaging buffer and injected into the flowcell for TIRF microscopy.

### Cas9 Binding Location and Duration Characterization

Binding location characterization was performed using a custom-written automated procedure. First, we manually marked the beginning and end positions of each DNA molecule in the 488 nm channel. Next, we performed 2D Gaussian fitting of fluorescent spots in the 635 nm channel in the areas that were marked in the previous step. The procedure fits each Cas9–ATTO647N complex (red-fluorescent spot in TIRF images acquired under 635 nm wavelength excitation) to the two-dimensional (2D) Gaussian function with the help of the detection of clusters of interconnected pixels that have values above the manually set threshold. This protocol is similar to the previously published one.^29^ All procedures were written using Igor Pro (Wavemetrics, Inc.) and are available upon direct request to the authors. Both center coordinates (*x* and *y*) were recorded for each detected fluorescent spot that had fitting error of all parameters below 60% from the parameter value. We mainly detected individual stable binding events of various durations without any diffusion characteristics. After fitting, we manually examined the fitted data and selected algorithm-suggested interconnected fit points (stable binding states) that contained more than five points (duration of at least 0.5 s) in them. This analysis allowed us to extract Cas9 binding-state durations (i.e., dwell times) and correlate them with the position on the DNA substrate.

## Results and Discussion

To utilize the DNA Curtains platform for complex protein–NA interaction studies, it is required to obtain double-tethered DNA molecule arrays with a defined orientation. Therefore, in this work, we upgraded the existing Soft DNA Curtains platform^14^ and further optimized its fabrication method by introducing several new steps that made the platform more stable and more controllable.

### Optimization of DNA Array Fabrication

**Figure 1:**
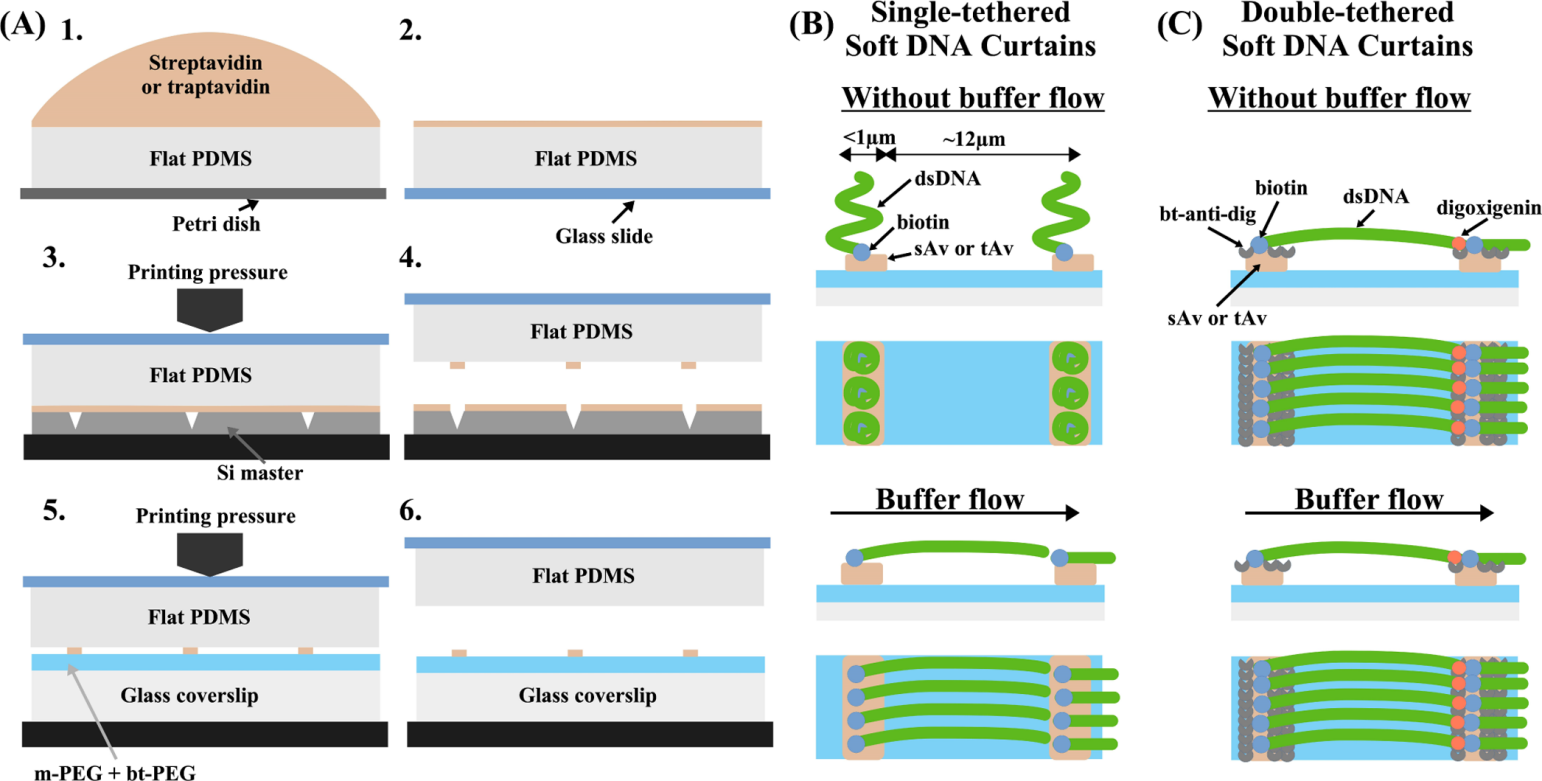
Diagram explaining the main steps of Soft DNA Curtains fabrication. (A) Diagram of protein lift-off microcontact printing (μCP): (1) inking of a planar PDMS stamp with streptavidin (sAv) or traptavidin (tAv) ink; (2) removal of the protein solution with the pipette, washing with buffer and ultrapure water, and drying of PDMS under a stream of N_2_ gas; (3, 4) selective subtraction (lift-off) of sAv (or tAv) by contacting the inked PDMS with the Si master under pressure applied by the portable printing device (PPD, indicated by the black arrow); (5, 6) μCP under pressure applied by the PPD of sAv or tAv onto a glass coverslip modified with the methoxy- and biotin-PEG mixture. (B) Diagram of single-tethered Soft DNA Curtains illustrating immobilization and alignment of biotin (bt)-labeled DNA on the fabricated sAv (or tAv) templates. (C) Diagram of double-tethered Soft DNA Curtains illustrating immobilization and alignment of bt- and digoxigenin (dig)-labeled DNA on the fabricated sAv (or tAv) templates via biotin–sAv/tAv and dig–anti-dig interactions.

Here, we expanded our previous work^14^ and demonstrated that lift-off μCP patterned sAv or, as we show here later, tAv protein templates (Figure 1A) can be employed for the self-assembly of biotin-labeled DNA molecules in the flowcell on the glass surface (Figure 1B). In principle, the design of the template ensures the distribution of biotinylated DNA molecules on predefined narrow protein line-features fabricated on the modified glass coverslip, which is otherwise resistant to nonspecific protein interactions. Application of the buffer flow pushes the DNA molecules through the flowcell channel while their biotinylated ends remain tethered. Also, the design of the template is such that the line-spacing distances of templates are sufficiently long and avoid overlapping of DNA molecules immobilized on the neighboring line-features. Such protein array templates can be considered as a soft functional element, and therefore we term our platform the Soft DNA Curtains.

First, to achieve protein array templates allowing the desired distribution of biotinylated DNA molecules, we fabricated Si masters using the previously described procedure^14^ with line widths ranging from ∼200 nm to 1 μm and 12 μm line-spacing corresponding to ∼75% of the mean extension of λ DNA.^6^ The dimensions of Si masters’ patterned area were from 0.5 × 0.9 to 2.5 × 1.2 mm^2^. The typical line-depth of the Si masters used in this work is ∼200 nm. Figure S1 shows the Si masters’ overall optical images and line-width and line-depth measurements using AFM. Table S1 summarizes the characteristics of Si masters that were measured by AFM.

**Figure 2:**
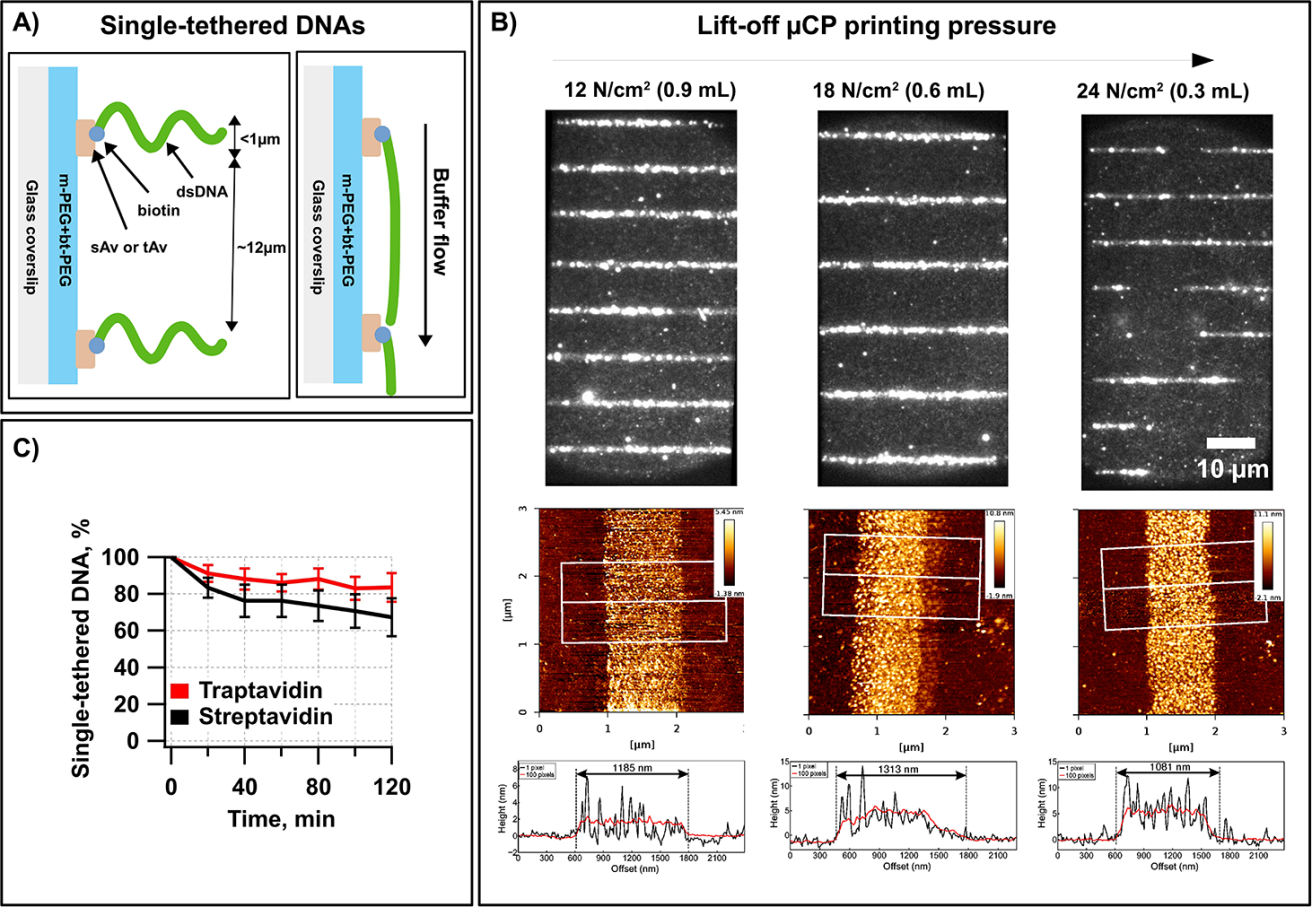
Optimization of DNA array fabrication. (A) Schematic of the single-tethered Soft DNA Curtains design showing the PEG monolayer on a glass coverslip and printed streptavidin (sAv) or traptavidin (tAv) line-features, which enables specific one-end immobilization of the biotinylated λ DNA (48.5 kb) molecules. DNA molecules are tethered to the line-features and respond to a hydrodynamic force by extending parallel to the surface. (B) Effect of printing pressure (PP) on the quality of short DNA molecule arrays. The top panel shows TIRF images of 5 kb-long biotinylated DNA molecules stained with SYTOX green (SG), which were immobilized on the sAv line-features fabricated on a modified coverslip. PP is indicated above each image. The bottom panel shows AFM images and their line-profiles (obtained as 1 pixel-width section over the line in the middle and averaged over 100 pixel-width area, which is indicated by the white square) of the sAv line-features fabricated on the modified glass coverslip at the same pressure as the TIRF images. [sAv] = 0.02 mg/mL, Si master no. 1. (C) Stability test of single-tethered Soft DNA Curtains: λ DNA molecules immobilized on a sAv (and tAv) array template and stained with SG. Images were acquired every 20 min for a period of 2 h. In between acquisitions, there was no buffer flow applied. During the acquisition, 20 frames were acquired at a buffer flow of 1 mL/min and 20 frames without the flow. The graph shows the average number of single-tethered DNA molecules that extended to the full length. The average was taken over line-features, and the error bars represent SD. Si master no. 3.

To improve the patterning reproducibility and to control the PP in the lift-off μCP, we built the PPD, which is similar to the previously published device.^30^ However, our PPD was assembled from commonly used parts in an optics laboratory and does not require sticking of the PDMS stamp to the moving piston (Figure S2). In addition to that, our PPD employs PDMS stamp attachment to the glass slide surface, which helps keep the stamp flat (Figures 1A and S2B). It is worth noting that a similar effect (PP vs protein array quality) could be achieved by changing the lift-off μCP printing time, but that would tremendously increase its duration. In our experiments, the pressure applied by this easy-to-use and relatively simple device ranged from ∼12 to ∼26 N/cm^2^. To keep it simple, instead of N/cm^2^, we chose to report the PP in terms of the position of the syringe 3 piston (Figure S2C). We calibrated this value, and the results are given in Figure S3. To test the quality of sAv line-features printed using PPD on the m-PEG/bt-PEG (10:1 w/w) modified glass coverslip surface, we immobilized 5 kb-long biotinylated double-stranded DNA (dsDNA) (Figure 2A). TIRF images showed that DNA molecules mainly immobilized on the sAv line-features, but their density was dependent on the applied PP (Figures 2B and S4A). Quantitatively, the best results were obtained with a PP of 0.6 mL (Table S2). We noticed that at 0.45 mL and especially at 0.3 mL PP DNA immobilization on the line-features was poor and lines became discontinuous. We observed a similar effect for other Si masters (no. 4, no. 6) with different line widths (Figure S4 B,C). This could be the result of either PDMS touching the bottom of the inscribed lines in the Si master during the printing procedure or some kind of pressure-induced inactivation of sAv. To test the first possibility, we performed AFM imaging of sAv lines printed with distinct pressures on the modified coverslip. Results of these measurements showed no evidence of either line breaks or line-width change (Figure 2B). Therefore, we concluded that too high PP (starting at 0.45 mL) inactivates a fraction of sAv on the surface, which results in reduced binding of biotinylated DNA, albeit PP values (<25 N/cm^2^, i.e., 2.5 bar) are far too low than that used for protein denaturation studies, typically 500 bar.^31−33^

Another parameter that we assessed to optimize the immobilization of biotinylated DNA molecules was the sAv concentration during PDMS elastomer inking under a constant PP of 0.6 mL. In these experiments, the sAv ink concentration ranged from 0.013 to 0.027 mg/mL. We performed lift-off μCP with sAv ink, assembled the flowcell, and immobilized the biotinylated 5 kb-long DNA molecules. The acquired TIRF images showed similar results as previously observed;^14^ the highest quality protein templates were fabricated at the moderate sAv concentration of 0.017 mg/mL (Figure S5 and Table S2). The optimal range of sAv concentration was rather narrow since concentrations that were 52% higher or lower than the optimal concentration immediately gave worse results. The obtained optimal sAv ink concentration under 0.6 mL of PP is similar to the optimal sAv concentration without applied PP.^14^ However, in contrast to the manual lift-off μCP performed by hand without application of pressure, the PPD device allows production of a consistent and high-quality protein template across the entire patterned area (Figure S6). This is the main advantage of lift-off μCP using PPD in comparison to the manual procedure.

In our previous work, we showed that the number of single-end-tethered biotinylated λ DNA molecules decreased slowly over time, with a half-life of >2 h,^14^ in good agreement with the expectations for a high-affinity biotin–sAv interaction. However, a recently developed superstable variant of sAv, called traptavidin,^15^ should allow us to observe immobilized biotinylated DNA molecules for an even longer period of time. We verified our tAv functionality (see the SI file and Figure S7) and then tested whether tAv is suitable for fabrication of the fixed DNA molecule arrays. For these experiments, we used the lift-off μCP with a variable tAv concentration, which ranged from 0.015 to 0.06 mg/mL, at a constant PP of 0.6 mL. Once line-features were formed and the flowcell was assembled, we immobilized biotinylated 5 kb-long DNA molecules. TIRF images showed that the optimal tAv concentration was ∼0.03–0.02 mg/mL (Figure S8C,D). Both at lower and higher concentrations than the optimal, we observed more DNA bound in the interline areas or lower DNA density on line-features (Figure S8A,B,E). This visual inspection was also well-reflected by the quantitative QF-based characterization (Table S2). However, the absolute density of DNA molecules immobilized on the line-features seems to be lower than that with sAv, but this could be rationalized by the different activity of tAv, which requires a higher concentration of DNA molecules to achieve similar densities. We also observed a similar effect of PP on the printed tAv line-features as for sAv (Figure S8F–H). At higher PP, the printed lines became discontinuous and showed a lower number of bound DNA molecules. Here, we decided to use the same concentration of sAv and tAv for the sake of consistency.

To test whether tAv indeed allows observing the immobilized DNA molecules for a longer period of time than sAv, we fabricated single-tethered Soft λ DNA Curtains on sAv (0.017 mg/mL) and tAv (0.03 mg/mL) line-features at a constant PP of 0.6 mL. Next, we performed the stability test of bound DNA molecules by performing the acquisition cycles of image series every 20 min. During these cycles, 20 frames were acquired with the 1 mL/min buffer flow and 20 frames without the flow. Between the acquisitions, there was no buffer flow applied. Acquired TIRF images showed that the number of full-length single-tethered DNA molecules anchored to the surface decreased slowly over time, with the half-life >2 h for both sAv and tAv (Figure S9). DNA molecules were dissociating slower from tAv than from sAv, and this is in good agreement with the expectation for the lower biotin dissociation constant of tAv.^15^ Namely, after 2 h of observation time, only ∼20% of the DNA molecules were dissociated from tAv line-features, while ∼40% of them were dissociated from sAv (Figure 2C). These results proved tAv to be better suited for the long-lasting experiments, which require observation of the same DNA molecules, and can be more beneficial for all types of DNA Curtains.

Finally, to test what forces our platform can sustain, we measured the shear flow extension of the single-tethered Soft DNA Curtains. The degree of DNA molecules’ extension increased at higher flow rates. The previous single-molecule studies have shown that the dynamic behavior of DNA can be mathematically modeled as the wormlike-chain model (WLC).^34−37^ We plotted the relative mean extension of the DNA molecule as a function of flow velocity and fitted that to an expression describing the WLC of DNA (Figure S9E). The data were well-fitted to the model, and we were able to exert forces ranging up to approximately 4 pN. We note that at higher flow rates (e.g., 3 mL/min, >4 pN) DNA molecules were still immobilized on the line-features. Thus, our platform is robust and could be used under different flow rates without loss of DNA molecules.

### Assembly and Characterization of Double-Tethered Soft DNA Curtains

**Figure 3:**
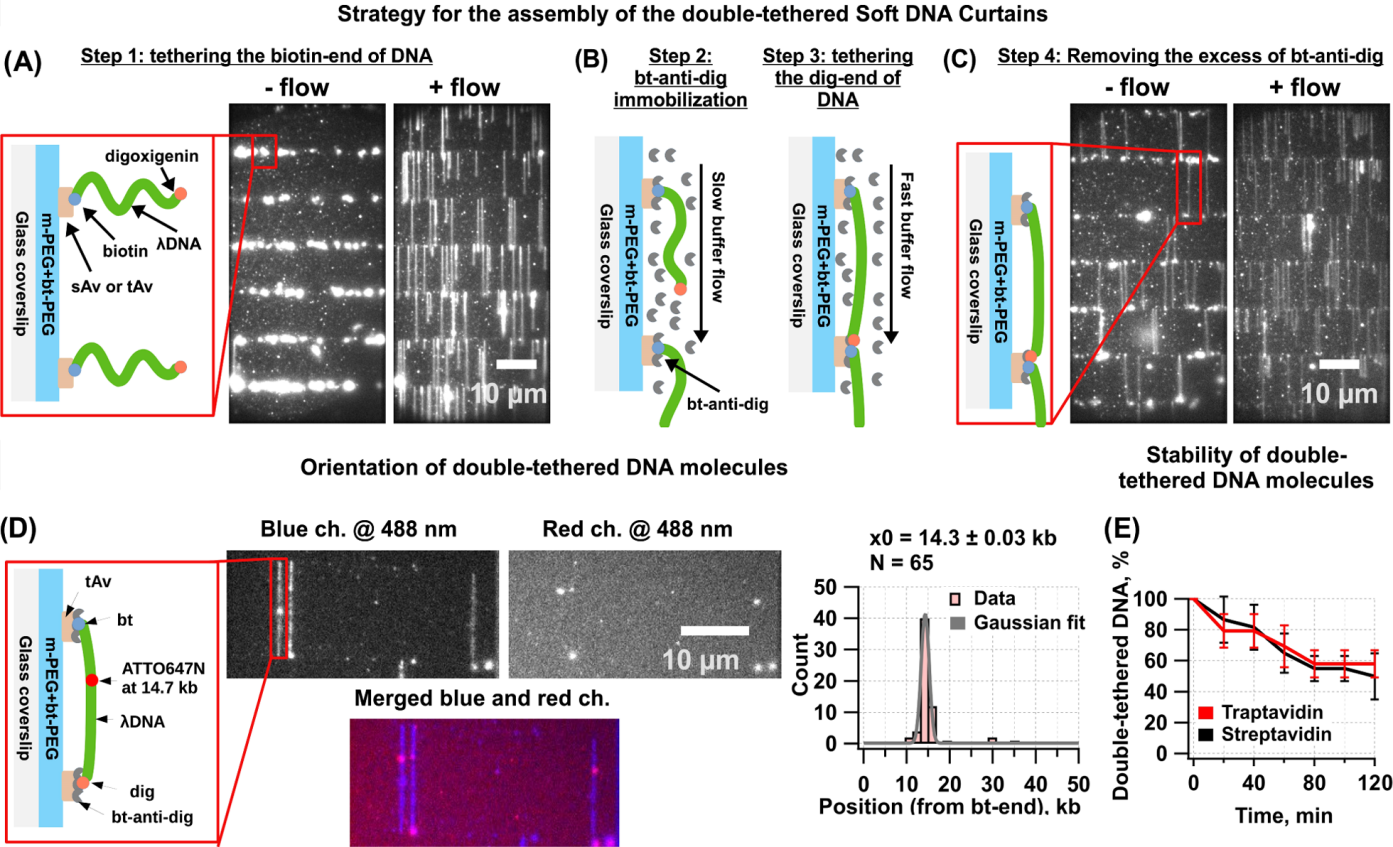
Double-tethered Soft DNA Curtains with a defined orientation. (A–C) Steps employed for assembly of the oriented double-tethered DNA Curtains. (A) Cartoon illustrating the printed traptavidin (tAv) line-features that enable specific one-end immobilization of the biotin-λ DNA-digoxigenin (bt-λ DNA-dig, 48.5 kb) molecules. TIRF images show that in the absence of the buffer flow (−flow), DNA molecules are aligned to the line-features. They are responding to a hydrodynamic force (+flow) by extending parallel to the surface. (B) To tether the dig-labeled end of bt-λ DNA-dig, continuous slow flow of the buffer containing biotinylated anti-dig (bt-anti-dig) antibody is applied and DNA molecules are dragged slightly but do not reach the neighboring line-feature. In the next step, the buffer flow rate is increased and the dig-labeled DNA ends encounter the neighboring line-feature, which is now covered with the bt-anti-dig, and the dig-labeled ends become anchored through the dig–anti-dig interaction. (C) Cartoon illustrating the double-tethered bt-λ DNA-dig molecule after dig-labeled end tethering. TIRF images show that DNA molecules that remain stretched without buffer flow (−flow) were successfully both-end-tethered. (D) Characterization of double-tethered DNA molecules’ orientation. Cartoon illustrating the DNA immobilization strategy and internal ATTO647N tag, which was located at 14 711 bp from the bt-end DNA. TIRF images showing SG-stained DNA molecules in the absence of buffer flow. Excitation wavelength and emission channel are indicated above each image. Histogram showing the distribution of ATTO647N locations that were determined by fitting the images to 2D Gaussian functions. Si master no. 8. (E) Stability test of double-tethered Soft DNA Curtains: λ DNA molecules immobilized on a sAv (and tAv) array template and stained with SG. Images were acquired every 20 min for a period of 2 h. During the whole experiment, there was no buffer flow applied. The graph shows the average number of double-tethered DNA molecules. The average was taken over line-features, and the error bars represent SD. Si master no. 8.

The patterns of our platform utilize tAv functional elements, and an overview of the general design is presented in Figure 1C. The biotinylated DNA end is first tethered on the protein line-features on the modified glass coverslip surface in the flowcell. In the absence of the buffer flow (hydrodynamic force), the molecules are distributed on the line-features but lie outside the penetration depth of the evanescent field (Figure 3A). Application of the buffer flow pushes the DNA through the flowcell channel while biotinylated DNA ends remain tethered. The other end of λ DNA was modified with the dig to tether it onto the neighboring protein line-feature. The line-spacing distance was optimized for the length of the λ DNA. The line-features themselves are designed to represent a sufficiently large surface, which can be coated by bt-anti-dig (for functionality verification of bt-anti-dig, see the SI file and Figure S10). When biotinylated DNA molecules are immobilized on the line-features, we apply slow flow of the buffer containing bt-anti-dig (Figure 3B). This step allows us to coat the protein line-features with the bt-anti-dig. In the next step, the immobilized DNA molecules are stretched by increasing the buffer flow rate. The dig-modified ends of DNA molecules should bind the antibody-coated line-features. This strategy allows us to hold DNA molecules stretched parallel to the surface even when no buffer flow is applied in the flowcell (Figure 3C).

Line-feature width of the tAv patterns was assessed to optimize the assembly of the double-tethered Soft DNA Curtains. Three different patterns of tAv were fabricated on the separate coverslips using Si masters no. 4, no. 5, or no. 6 at a PP of 0.6 mL. The double-tethered Soft DNA Curtains were assembled on the patterned coverslips as described above (Figure 3). The anchoring efficiency was tested for tAv patterns made with variable line widths but constant line-spacing (i.e., ∼12 μm). As expected, the wider lines allowed us to achieve more efficient anchoring. Percentages and densities (average number of both-end anchored DNA molecules per line-feature) of both-end anchored DNA molecules are presented in Table S3. Approximately 79% of the anchored DNA were double-tethered with 1 μm-wide, 74% with 500 nm-wide, and 71% with 350 nm-wide line-features. More apparent differences were observed in densities of double-tethered DNA molecules. For the 1 μm-wide, 500 nm-wide, and 350 nm-wide line-features, the values were, respectively, ∼4.9, ∼3.6, and ∼1.6 double-tethered DNA molecules per line-feature. Thus, the thicker the line-feature, the higher the density of both-end-tethered DNA molecules.

Once line-feature width was optimized, the line-separation distance of the tAv patterns was assessed. Four different patterns of tAv were fabricated on the separate coverslips using Si masters no. 5, no. 7, no. 8, and no. 9 at a PP of 0.6 mL. The double-tethered Soft DNA Curtains were assembled on the patterned coverslips as described above (Figure 3). This time, the anchoring efficiency was tested for tAv patterns made with variable line-separation distances but constant line widths (i.e., ∼0.5 μm). As expected, the most efficient anchoring occurred with the 12 and 13 μm line-spacing distances. Percentages of both-end-anchored DNA molecules are presented in Table S4. Approximately 80, 74, 48, and 20% of the anchored DNA were double-tethered, respectively, with 13, 12, 11, and 14 μm separation distance line-features.

When line-width and line-separation distance were optimized, we tested the orientation of λ DNA fragments on the double-tethered Soft DNA Curtains using a fluorescent tag (ATTO647N at the position 14 711 bp) introduced asymmetrically at the specific position of the λ DNA. As mentioned above, we used differential chemistries (biotin on the left end, and dig on the right end) to tag the two ends of the DNA (Figure 3A–C). Therefore, molecules within the double-tethered curtains should be immobilized in a defined orientation. To confirm that the DNA was oriented correctly, we assembled double-tethered Soft DNA Curtains from the ATTO647N-labeled λ DNA (bt-λ DNA ATTO647N-dig) as described above (Figure 3D). The ATTO647N labels were present at a single location within the DNA molecules and aligned with one another. Their mean position was found to be 14.3 ± 0.03 kb (*N* = 65) from the biotinylated DNA end. This result coincided well with the expected location, and practically no ATTO647N labels were observed at other locations.

The stability of double-tethered Soft DNA Curtains should be lower than that of the single-tethered ones because here the limiting factor is the weaker dig–anti-dig interaction used to tether the second DNA end. To test whether this is valid, we fabricated double-tethered Soft λ DNA Curtains on sAv (0.017 mg/mL) and tAv (0.03 mg/mL) line-features at a constant PP of 0.6 mL. Next, we performed the stability test of bound DNA molecules by performing the acquisition cycles of image series every 20 min. During the whole experiment, there was no buffer flow applied. Results showed that the number of both-end-tethered DNA molecules decreased over time, with a half-life of ∼2 h for both sAv and tAv (Figure 3E). DNA molecules were dissociating at a similar rate from both tAv and sAv, and this is in good agreement with the expectation.

### Deployment of Double-Tethered Soft DNA Curtains for Visualization of Protein–Nucleic Acid Interactions

**Figure 4:**
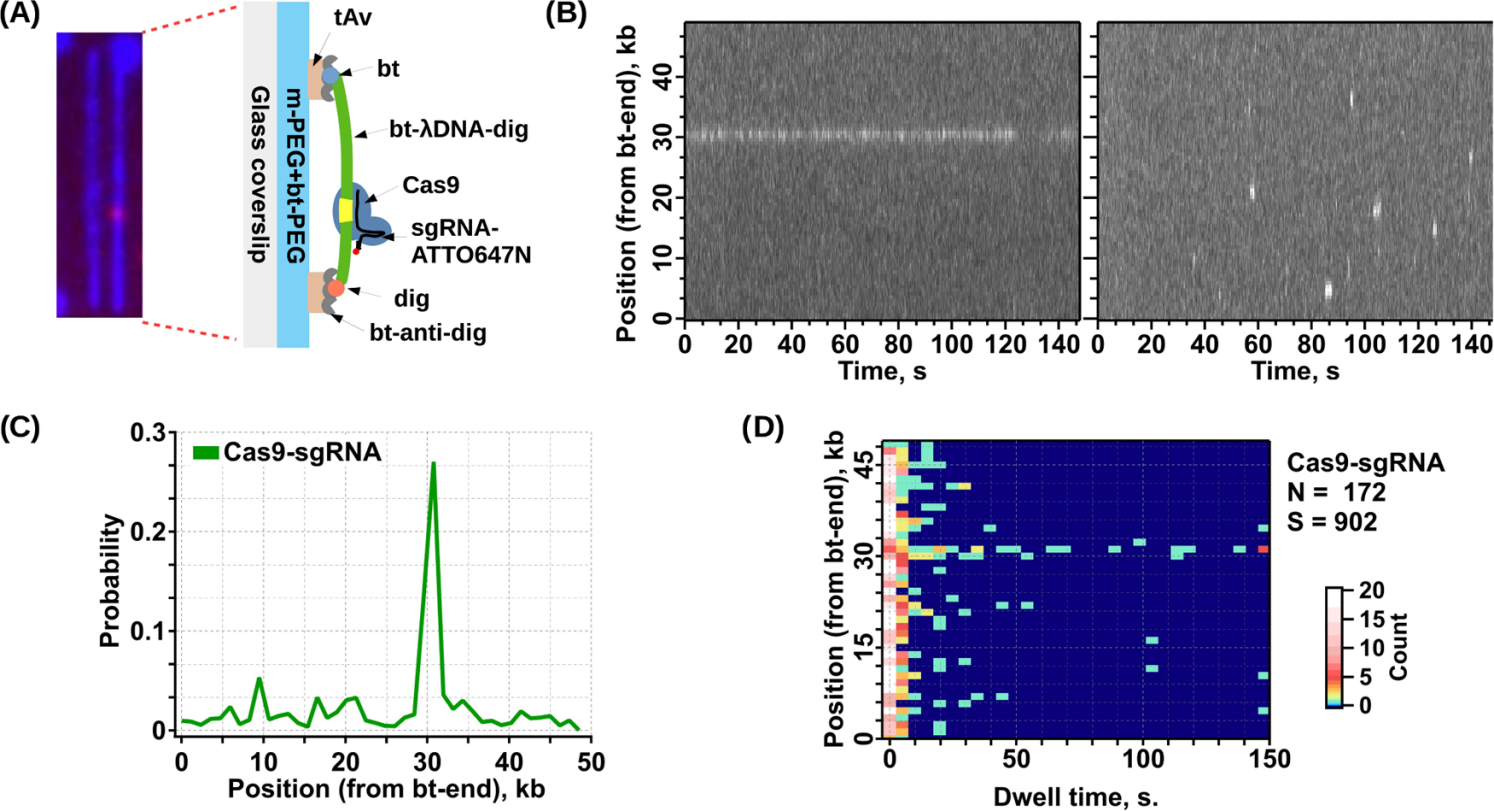
Double-tethered Soft DNA Curtains assay for binding location studies of the SpCas9 protein. (A) Merged blue and red channel TIRF images representing SG-stained λ DNA (blue) with bound SpCas9 (red). Schematic representation of the assay depicts SpCas9, which was programmed with ATTO647N-labeled crRNA-tracrRNA (Cas9–ATTO647N) targeting site of λ DNA located at 31.1 kb from the biotinylated DNA end. (B) Representative kymograms made from individual DNA molecules. (C) Histogram of SpCas9–ATTO647N binding events’ distributions determined by 2D Gaussian fitting. (D) SpCas9–ATTO647N binding position vs dwell time 2D histogram plot. The plot was made from 172 DNA molecules and contains 902 individual binding events. Color code represents the counts.

To demonstrate that double-tethered Soft DNA Curtains can be utilized to visualize protein–DNA interaction, we selected previously characterized Cas9 nuclease from the CRISPR-Cas system of *S. pyogenes*, which is involved in bacterial defense against foreign invading DNA and has been adopted as a genome editing tool.^38^ Soft DNA Curtains were assembled from bt-λ DNA-dig molecules and tAv line-features covered with the bt-anti-dig. The Si master no. 8 was used to fabricate the tAv line-features on a PEGylated coverslip at a PP of 0.6 mL. The Cas9 complex targeting double-tethered λ DNA 31.3 kb from the biotin-labeled end was fluorescently labeled with the ATTO647N-oligonucleotide that was complementary to the tracrRNA 5′-end (Figure 4A). The SpCas9–ATTO647N complexes were diluted to 0.2 nM concentration in a Mg^2+^-free buffer and injected into a flowcell. We decided to exclude Mg^2+^ ions to avoid DNA cleavage.

TIRF images show an example of double-tethered curtains with bound Cas9–ATTO647N, where DNA is colored blue and the protein is colored red (Figure 4A). This image represents a single 100 ms-long image taken from a 150 s video (the full-length video is available in the SI file). Two representative kymograms extracted from two different DNA fragments show long-lasting binding events occurring on the target site (31.3 kb) and short binding events occurring on the nontarget site (Figure 4B left and right, respectively). A binding profile of Cas9–ATTO647N was obtained from individual DNA molecules (Figure 4C). In total, 172 DNA molecules and 902 binding events of proteins were monitored during this experiment. As in Figure 4, the DNA-bound Cas9–ATTO647N demonstrated broad binding distribution along the full length of the λ DNA. However, in total, proteins spend more time at the target site (Figure 4C). There were more binding events on the left side of the DNA, which likely is dictated by the higher GC content. Binding events on the target had significantly longer dwell times (some of them lasted as long as the acquisition time of 150 s) than on nontarget binding, which lasted <20 s (Figure 4D). On average, target binding events lasted for ∼52 s and nontarget binding events lasted for ∼7 s (Figure S11). We note that this experiment was conducted in the absence of Mg^2+^ ions in the imaging buffer. Therefore, the obtained results suggest that Mg^2+^ ions are not essential for specificity of SpCas9 target recognition. Additionally, photobleaching control of Cas9–ATTO647N showed that this dye on average fluoresces for ∼173 s until full bleaching (Figure S12), limiting the acquisition of a complete data set for long dwell on-target binding events, which was shown previously to have lasted longer than 500 s.^38^ Nevertheless, these results suggest that without RNA–DNA heteroduplex formation, the Cas9 complex is able to quickly dissociate from the DNA, utilizing an efficient DNA target search mechanism, providing direct evidence that the double-tethered Soft DNA Curtains can be utilized to visualize the DNA and fluorescently labeled proteins’ interaction at the SM level.

## Conclusions

The oriented double-tethered DNA Curtains allow intuitive and simple parallel examination of hundreds of SMs in a single TIRF experiment. Our developed Soft DNA Curtains platform is an alternative to well-established DNA flow-stretch assays, which may be attractive for some SM laboratories.^39^ In this work, we made several significant improvements compared to a previously published study.^14^ First, we showed that it is possible to fabricate oriented and aligned Soft DNA Curtains using a high-quality protein-template-directed assembly of biotinylated DNA molecules and hydrodynamic force. Second, we showed by the Cas9 binding experiment that our oriented double-tethered Soft DNA Curtains are suitable for visualization and characterization of individual NA-interacting protein studies. In addition to that, we demonstrated how to make semiautomated PPD that simplified the protein lift-off μCP procedure and made it more reproducible and easier to control. To our best knowledge, this is the first time that such a home-built device was employed for a protein lift-off μCP procedure. Here, we adopted this PPD to show how to press the PDMS stamp during the protein lift-off μCP procedure to obtain the best-quality printed protein line-features. Finally, we demonstrated PP-dependent inactivation of sAv and tAv, which was independent of the line-feature dimensions inscribed in the Si master. We believe that each of the optimizations and improvements described in this work will make the Soft DNA Curtains platform more attractive for SM studies.

## Supporting Information Available

TIRF images show an example of double-tethered curtains with bound Cas9–ATTO647N under 635 nm wavelenght excitation (Movie 1) (AVI)
Additional methods; List of parameters of Si masters use; overview of Si masters used; portable printing device (PPD); syringe piston position and the printing force calibration curve of the portable printing device; list of the quality factors (QF) obtained by changing the printing pressure (PP); printing quality across the full area of the template; Image1 (PDF)

## Supplementary Material

la1c00066_si_002.avi
