# Supplementary material for: Oriented Soft DNA Curtains for Single-Molecule Imaging
Source: Langmuir. 2021 Mar 9;37(11):3428–37. doi: 10.1021/acs.langmuir.1c00066 (PMC8280724; doi:10.1021/acs.langmuir.1c00066)
Supplement: Supplementary file 2 — la1c00066_si_003.pdf [file la1c00066_si_003.pdf]

Supporting Information

Oriented Soft DNA Curtains For Single Molecule Imaging

Aurimas Kopūstas,[†‖] *Šarūnė Ivanovaitė,*[†] *Tomas Rakickas,*[‡] *Ernesta Pocevičiūtė,*[‖§] *Justė Paksaitė,*[‖] *Tautvydas Karvelis,*[‖] *Mindaugas Zaremba,*[‖] *Elena Manakova,*[‖] *Marijonas Tutkus.*[†‖*]

[†]Departments of Molecular Compound Physics, [‡]Nanoengineering, and [§]Functional Materials and Electronics, Center for Physical Sciences and Technology, Savanorių av. 231, Vilnius LT-02300, Lithuania.

[‖]Institute of Biotechnology, Life Sciences Center, Vilnius University, Saulėtekio av. 7, LT-10257, Vilnius, Lithuania.

*e-mail: marijonas.tutkus@ftmc.lt

Portable printing device (PPD). The basic principles of our PPD device (Figure S2) are similar to the previously published one,[1] and it was assembled from commonly used parts in an optics laboratory. As the Figure S2A shows, firstly we placed the Si master on a silicone rubber sheet attached to a glass slide (microscope slide, 1 mm thickness, Heinz Herenz Hamburg), which was glued to a base plate (3BP4-02, Standa) using a piece of double sided sticky tape (9088-200, 3M). We made holes in the centers of the silicone rubber sheet, the glass slide, and the double-sided sticky tape. To keep the Si master fixed on the silicone rubber sheet, we sucked out the air through this hole. For this purpose, we glued a nanoport (10-32, IDEX Health & Science) to the center of the under side of the glass slide and connected it to a 2 mL syringe (syringe 1 in the Figure S2A, Omnifix, LY21.1 Carl-Roth) via a 30 cm long tubing (a suitable tubing clamp was installed to clamp the tube after the air was sucked out). After this step, we attached a protein-coated PDMS elastomer with a protein ink monolayer flat-side-up onto the glass slide using double-sided sticky tape (Figure S2B). Next, we held the glass slide with tweezer and positioned the PDMS elastomer facing inked-side-down onto the Si master. To apply printing pressure, we made a system of two tubing-connected syringes (Figure S2C, 1 mL Omnifix-F Solo Single-use syringe, 9161406V, Braun). First, we fitted the first syringe (syringe 2 in the Figure S2C) into the center hole of two connected base plates (3BP4-02, Standa). Second, we filled the syringes and the tubing with tap water and fixed the syringes to the 15 cm long tubing (228-0703, VWR) using tube clamps (EX52.1, Carl-Roth) and 3 cm long adaptor tubings (N874.1, Carl-Roth). These adaptor tubings were placed on the ends of the 15 cm long tubing and made the contact with the syringe stronger. Once the system was assembled, we mounted it onto mounting posts (3MP-25+3AH6-4, Standa) using suitable screws (M6, 3 cm long). Finally, we applied the printing pressure to the PDMS elastomer using the second syringe (syringe 3 in the Figure S2C) and controlled the printing pressure by visually inspecting the volume scale on the second syringe.

Printing force measurements. To measure the actual printing force applied with the device, we employed a force sensitive resistor (FSR 400 Series Round, 5.08 mm^2 active area, 0.35 mm nominal thickness, Interlink Electronics, Inc.), which was placed in between syringe 2 and the glass slide holding the PDMS elastomer (Figure S2C). For this measurement we followed previously described procedure.[2,3] One leg of the FSR device was connected to the +5 V (V_{in}) output port of the Arduino Uno card and the other leg was tied to a measuring resistor (R_m = 10 kΩ) in a voltage divider configuration (i.e. one end was connected to ground and the other to the analog input port of the Arduino Uno card). The output voltage (V_{out}), which was monitored using the Arduino Uno card, increased with increasing force. To calculate the actual printing pressure in Newtons per square centimeter (N), we used the following equations for resistance (R) and conductance (C). Note, our PDMS elastomer had 5 x 5 mm^2 dimensions, therefore to get the force in N/cm^2 we introduced multiplication factor 4 into equation 3 (multiplication by the factor of 4 is embedded in the equation). Finally, we used conversion factor (k) obtained from the calibration curve of this sensor to calculate the actual force.[3] The obtained syringe position – printing pressure calibration curve is shown below in Figure S3.

$$R = \frac{(V_{in} - V_{out}) \cdot R_m}{V_{out}} \ (1);$$

$$C = \frac{1}{R} \ (2);$$

$$N = C \cdot k \cdot 4 \ (3)$$

Functionality of traptavidin. We made sure that our tAv was indeed functional and bound biotinylated 5 kb long DNA molecules. For that purpose we acquired TIRF images at 488 nm wavelength excitation and they showed no DNA binding on the surface modified by only m-PEG (Figure S7A), while the m-PEG/bt-PEG mixture modified surface was densely covered with DNA molecules (Figure S7B). Both surfaces were incubated with 0.02 mg/mL tAv, washed, incubated

with 5 kb long DNA, washed and then stained with SG. These results verified functionality of the surface bound tAv molecules.

Functionality of biotinylated antibodies directed against digoxigenin. We prepared biotinylated anti-dig antibodies by conjugating them with Biotin-PEG4-NHS ester. Functionality of these bt-anti-dig conjugates was tested on a surface. The bt-anti-dig were immobilized on a PEGylated surface (10% bt-PEG) covered with sAv. Next, the singe-end dig-labeled λ DNA (dig-λ DNA) molecules were immobilized, stained using SG, and visualized using TIRF microscopy at 488 nm excitation without (Figure S10A) or with buffer flow (Figure S10B). These images showed mainly one-end tethered DNA fragments, suggesting successful anti-dig and dig-λDNA interaction. No binding of dig-λ DNA was observed on the PEGylated surface (10% bt-PEG) covered with only sAv.

Table S1: List of parameters of Si masters used in this work.

Name of Si master	Line-width [nm]	Line-depth [nm]	Size of the patterned area [cm^2]	Comments
1	~1000	~320	2.5 x 1.2	Variable line-spacing ranging from 9.4 to 14.4 µm.
2	~430	~200	0.7 x 0.8	Variable line-spacing ranging from 12 to 14.5 µm.
3	~180; ~900	~200	1.3 x 1.1	Alternating line-width: first line thin and the following thick. Constant line-spacing of 12 µm.
4	~500	~300	0.5 x 0.9	Constant line-spacing of 12 µm.
5	~1000	~400	0.5 x 0.9	Constant line-spacing of 12 µm.
6	~350	~200	0.5 x 0.9	Constant line-spacing of 12 µm.
7	~500	~200	0.5 x 0.9	Constant line-spacing of 11 µm.
8	~500	~200	0.5 x 0.9	Constant line-spacing of 13 µm.
9	~500	~200	0.5 x 0.9	Constant line-spacing of 14 µm.

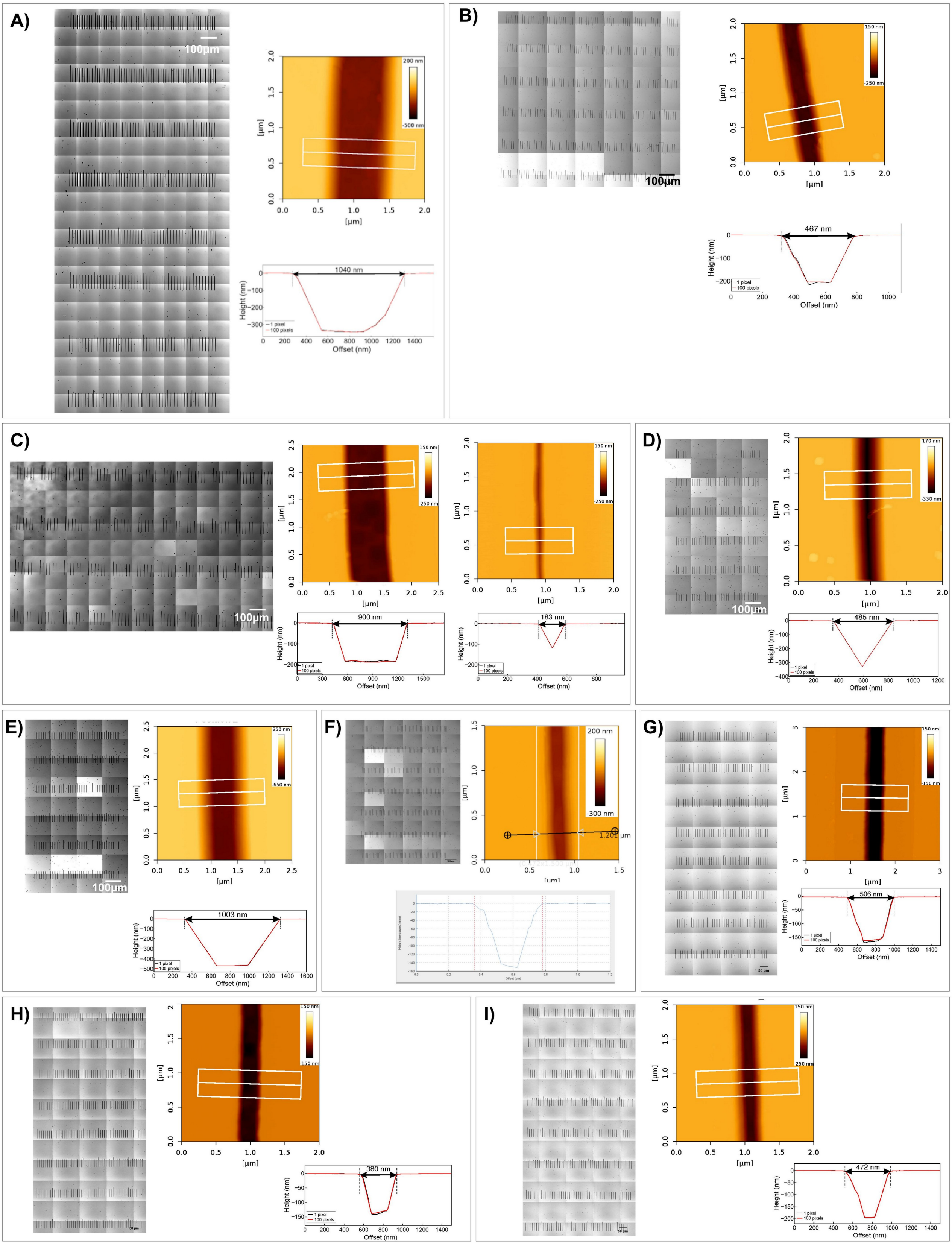

Figure S1: Overview of Si masters used in this work. **A – I)** On the left of each panel there is an optical image of the Si master #1 - #9 respectively after gold layer removal, on the middle there is an AFM image showing inscribed line profile and on the right bottom there are line-profiles (obtained as 1 pixel-width section over the line in the middle and averaged over 100 pixels-width area, which is indicated by the white square) taken on the AFM image. For panel **C)** only difference is that this Si master contains alternating thick (~900 µm) and thin (~180 µm) lines.

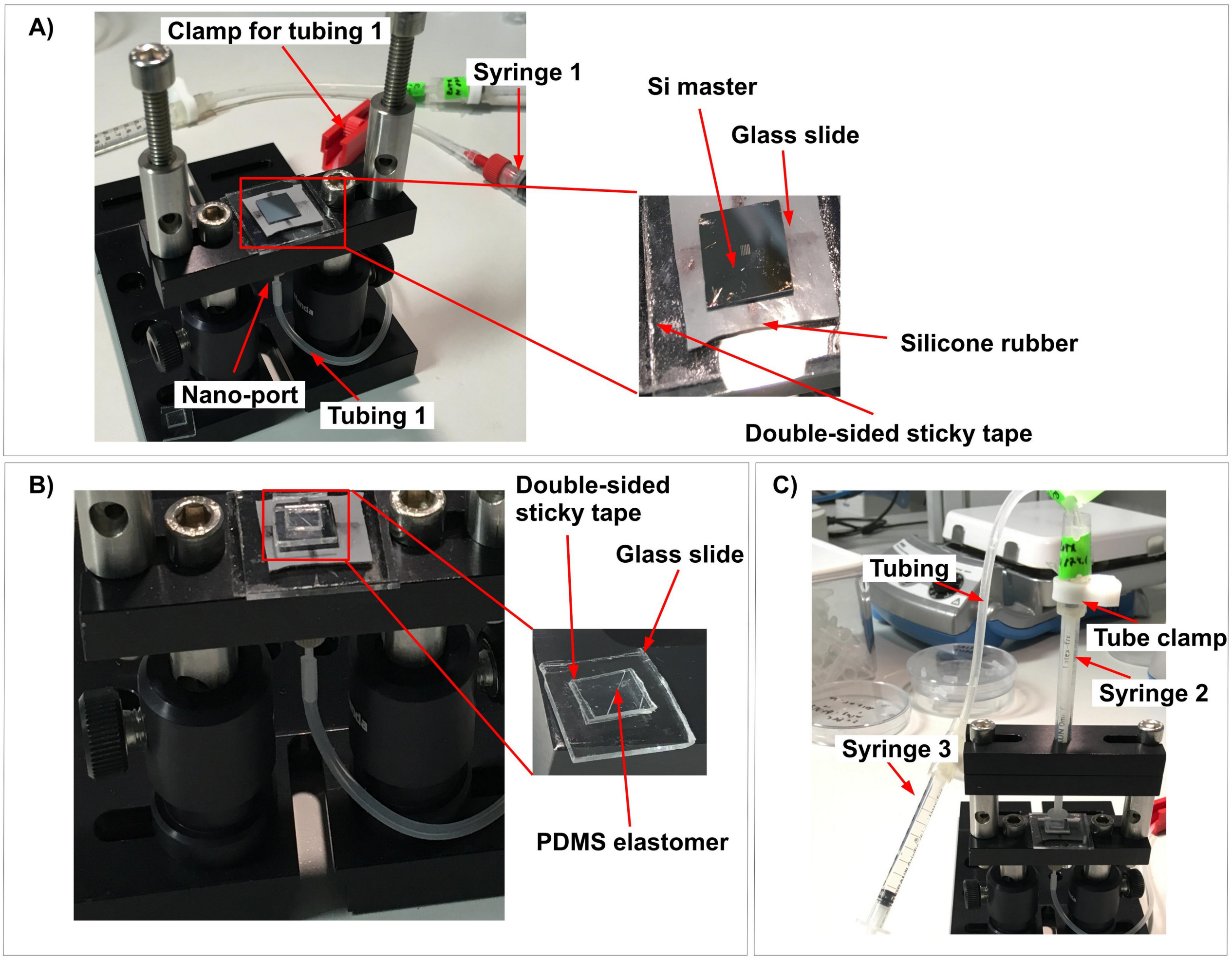

Figure S2: A portable printing device (PPD). **A)** Photograph of the PPD device with a Si master. **B)** The glass slide with attached PDMS elastomer was placed topside down onto the Si master manually using a tweezer. **C)** The system of two tubing-connected syringes was positioned above the glass slide with the PDMS elastomer, and printing pressure was applied by pressing the distant syringe (syringe 3). We controlled the printing pressure by monitoring the volume scale on the syringe 3.

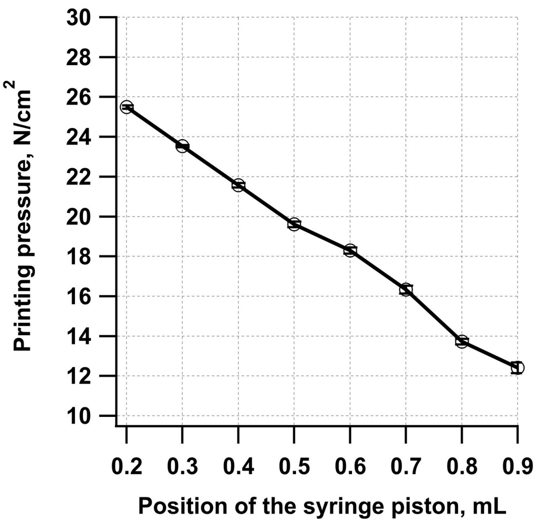

Figure S3: Syringe piston position and the printing force calibration curve of the portable printing device (PPD). Mean values (open circles) and SD (error bars) represents six measurements.

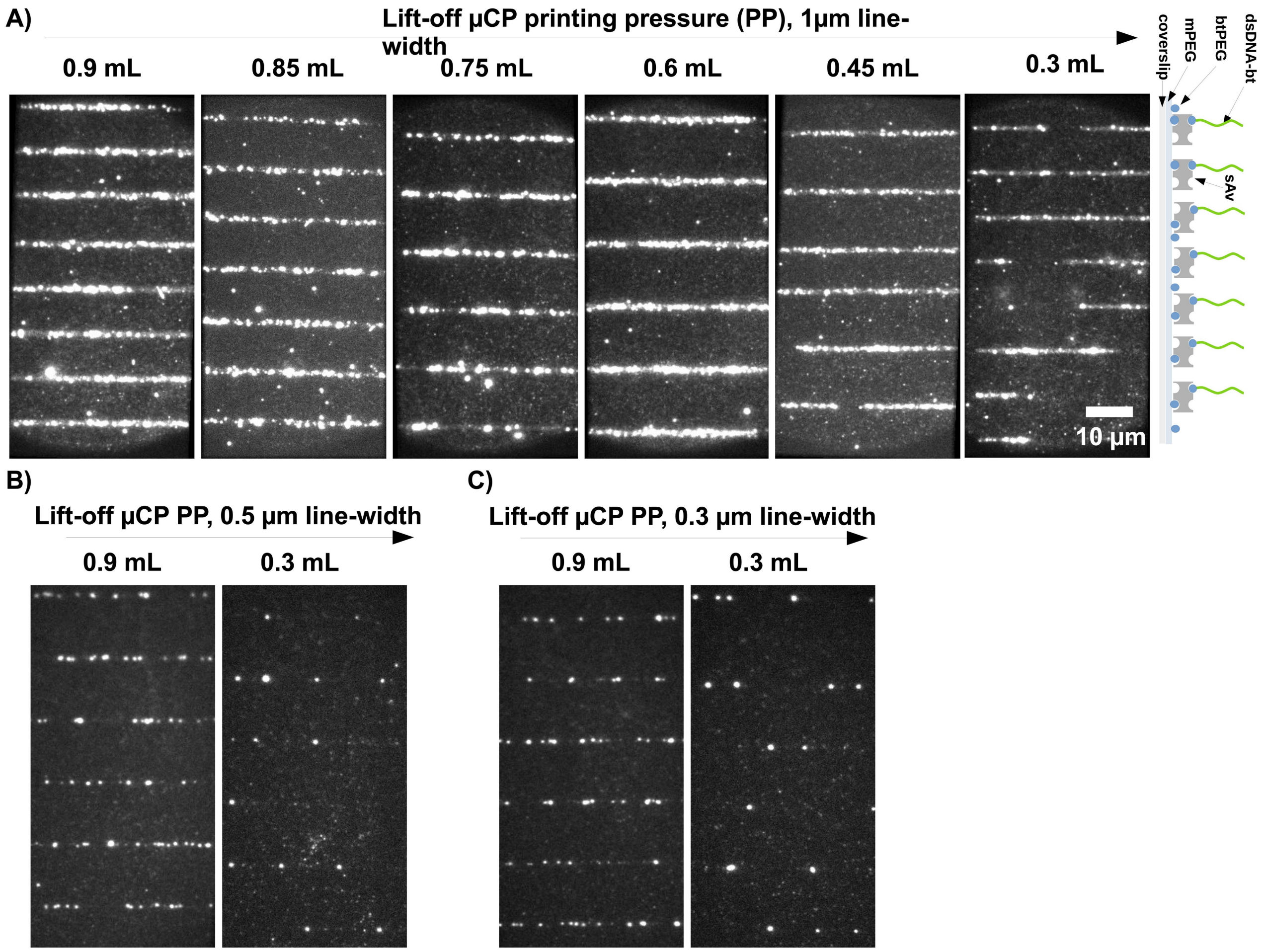

Figure S4: Characterization of short DNA molecule arrays and printing pressure (PP) effect on their quality. TIRF images of 5 kb long biotinylated DNA molecules stained with SYTOX green (SG), which were immobilized on the streptavidin (sAv) line-features fabricated on modified coverslip. A) Cartoon illustration on the right side depicts the immobilization scheme. The PP, which was varied from the low (0.9 mL) to the relatively high (0.3 mL), is indicated above each image. [sAv] = 0.02 mg/mL, Si master #1. B-C) Effect of PP (0.9 and 0.3 mL) with different line-width Si masters. B) Si master #6, and C) Si master #4, Images presented here were acquired at 488 nm wavelength excitation and averaged over 10 consecutive frames

Table S2: List of the quality factors (QF) obtained by changing the printing pressure (PP) of the lift-off µCP or streptavidin (sAv) and traptavidin (tAv) ink concentration during PDMS elastomer inking while keeping the PP constant. SEM – standard error of the mean.

PP [mL] at 0.02 mg/mL of sAv	〈QF〉 ± SEM	sAv concentration [mg/mL] at 0.6 mL PP	〈QF〉 ± SEM	tAv concentration [mg/mL] at 0.6 mL PP	〈QF〉 ± SEM
0.9	12 ± 2.2	0.013	14.22 ± 2.1	0.06	6.5 ± 1.03
0.85	13 ± 1.7	0.017	21.1 ± 3	0.04	5.1 ± 0.3
0.75	16.8 ± 3.5	0.027	12.7 ± 1.1	0.03	10.4 ± 0.4
0.6	17.7 ± 2.7			0.02	10.3 ± 0.8
0.45	14.7 ± 3.1			0.015	5.7 ± 0.9
0.3	5.8 ± 0.7				

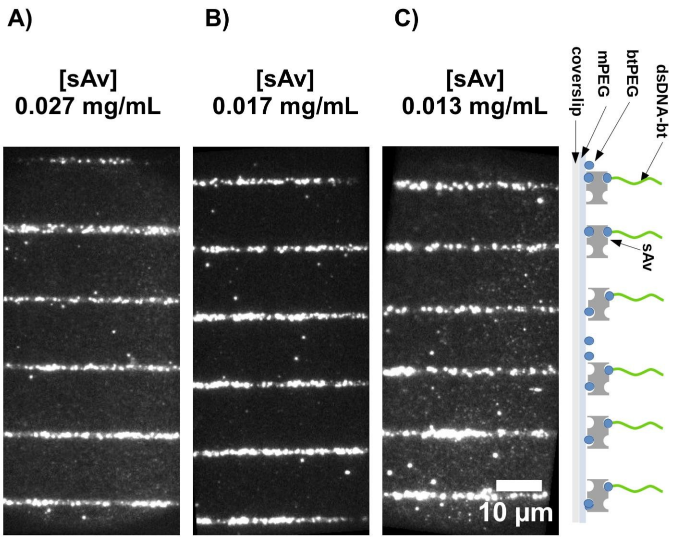

Figure S5: Characterization of short DNA molecule arrays and streptavidin (sAv) ink concentration effect on their quality at constant printing pressure (PP). **A-C)** TIRF images of 5 kb long biotinylated DNA molecules stained with SG, which were immobilized on the sAv line features fabricated on modified glass coverslip. Concentration of sAv ink: **A)** 0.027 mg/mL, **B)** 0.017 mg/mL, **C)** 0.013 mg/mL. Cartoon illustration on the right side depicts the immobilization scheme. PP = 0.6 mL, Si master #1, TIRF images were acquired at 488 nm wavelength excitation and averaged over 10 consecutive frames.

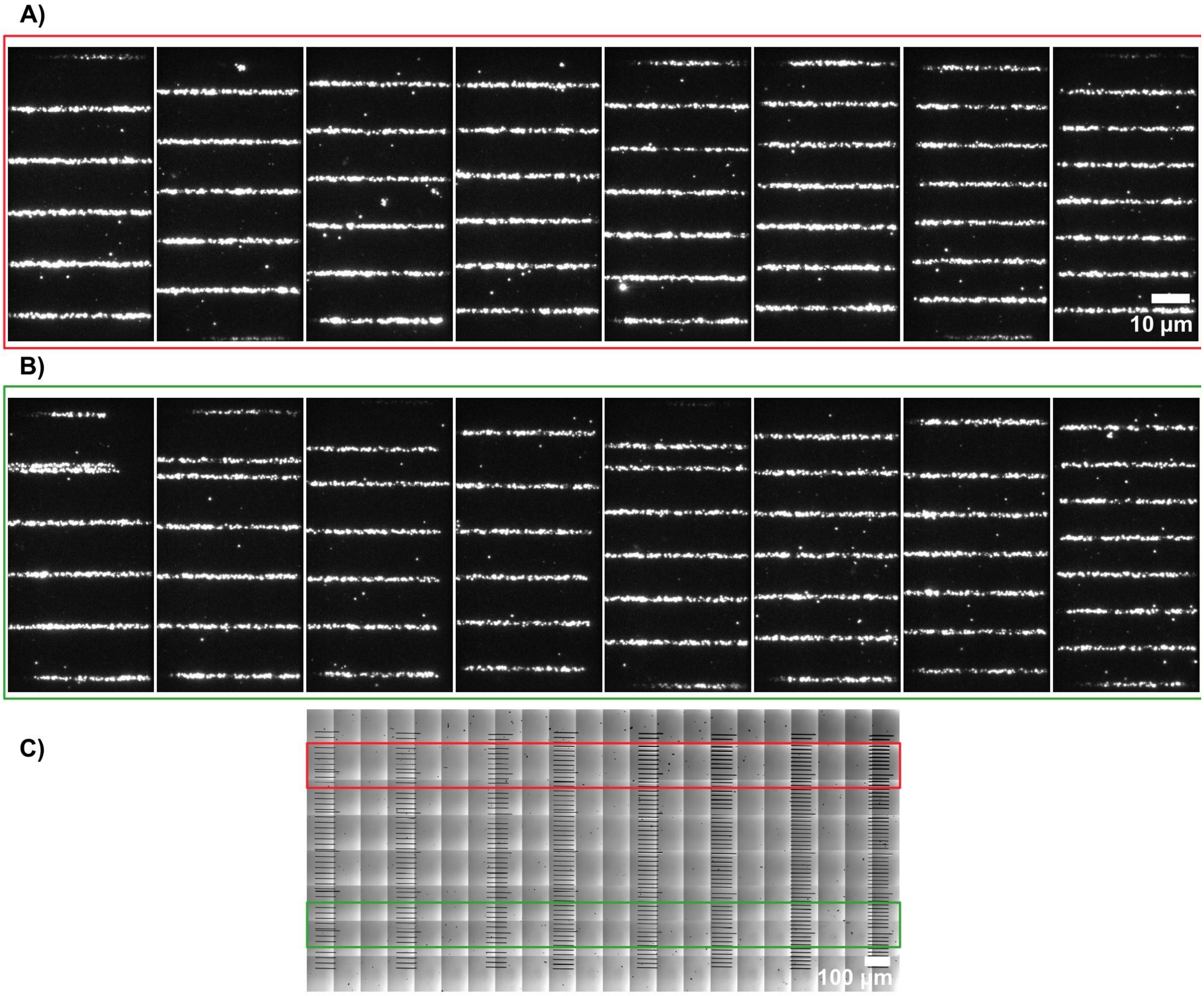

Figure S6: Printing quality across the full area of the template. **A-B)** TIRF micrographs of the single-tethered and SYTOX Green stained 5 kb DNA molecules immobilized on streptavidin template fabricated using Si master #1 and printing pressure (PP) equal to 0.6 mL. Excitation was at 488 nm wavelength and images are averages of 10 consecutive frames. **C)** Optical image of the Si master #1. Red and green squares represents region of interests for the **A** and **B** panel images.

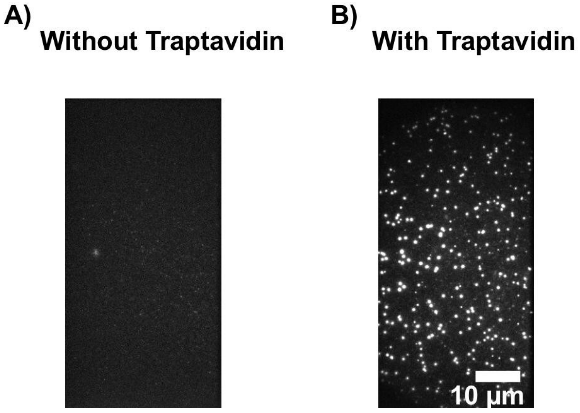

Figure S7: Functionality of surface immobilized traptavidin (tAv). The biotinylated 5 kb DNA molecules were immobilized on either **A)** bare or **B)** tAv covered (at 0.02 mg/mL) PEGylated (10% biotin-PEG) glass coverslip surface. DNA molecules were stained with the SYTOX Green, TIRF images represented here were acquired at 488 nm wavelength excitation and averaged over 10 consecutive frames.

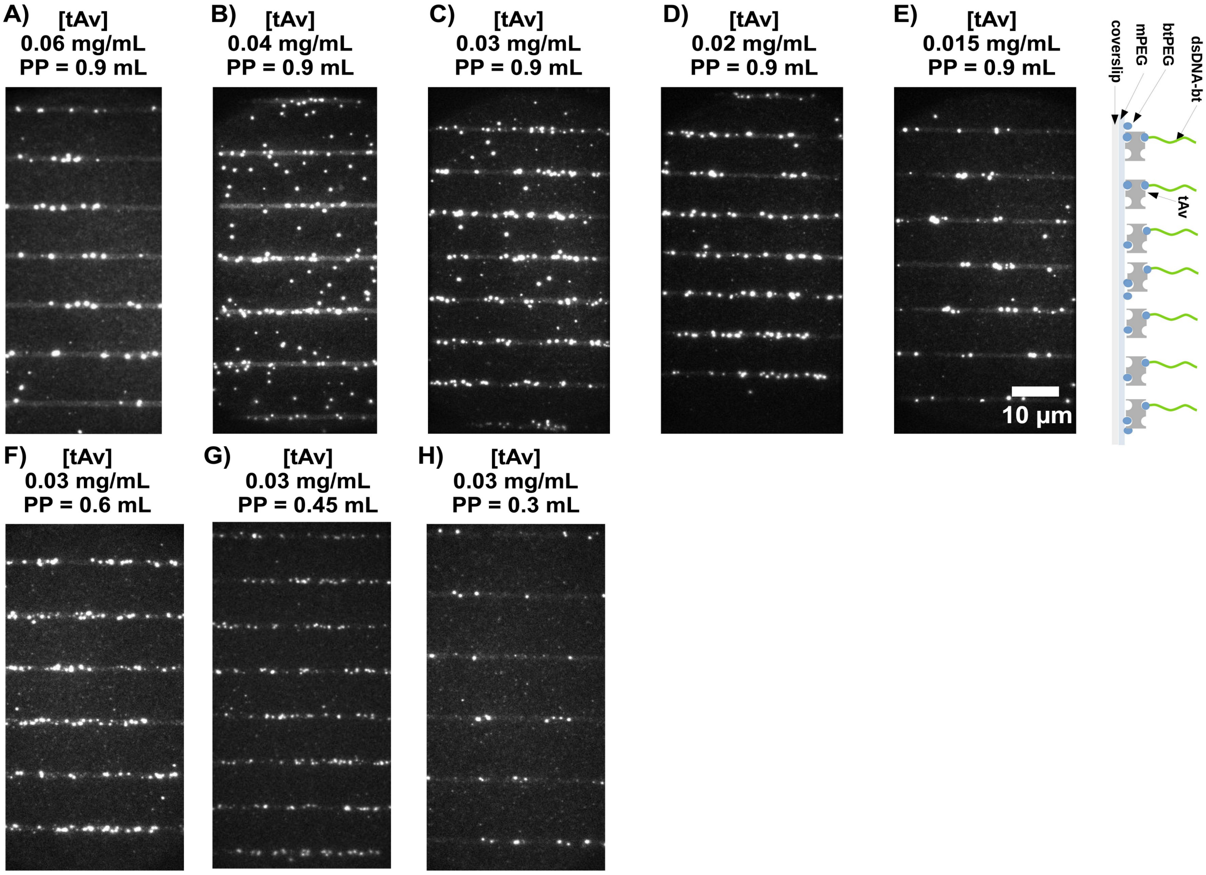

Figure S8: Characterization of short DNA molecule arrays and traptavidin (tAv) ink concentration effect on their quality and effect of printing pressure (PP). **A-E)** TIRF images of 5 kb long biotinylated DNA molecules stained with SYTOX green (SG), which were immobilized on the tAv line features fabricated on modified glass coverslip. Concentration of tAv ink: **A)** 0.06 mg/mL, **B)** 0.04 mg/mL, **C)** 0.03 mg/mL, **D)** 0.02 mg/mL, **E)** 0.015 mg/mL. Cartoon illustration on the right side of the panel **E** depicts the immobilization scheme. PP = 0.9 mL, Si master #1. **F-H)** Effect of PP on the quality of printed tAv line-features. Concentration of tAv ink: 0.03 mg/mL. The PP was: **F)** 0.6, **G)** 0.45, and **H)** 0.3 mL. TIRF images presented in this figure were acquired at 488 nm wavelength excitation and averaged over 10 consecutive frames.

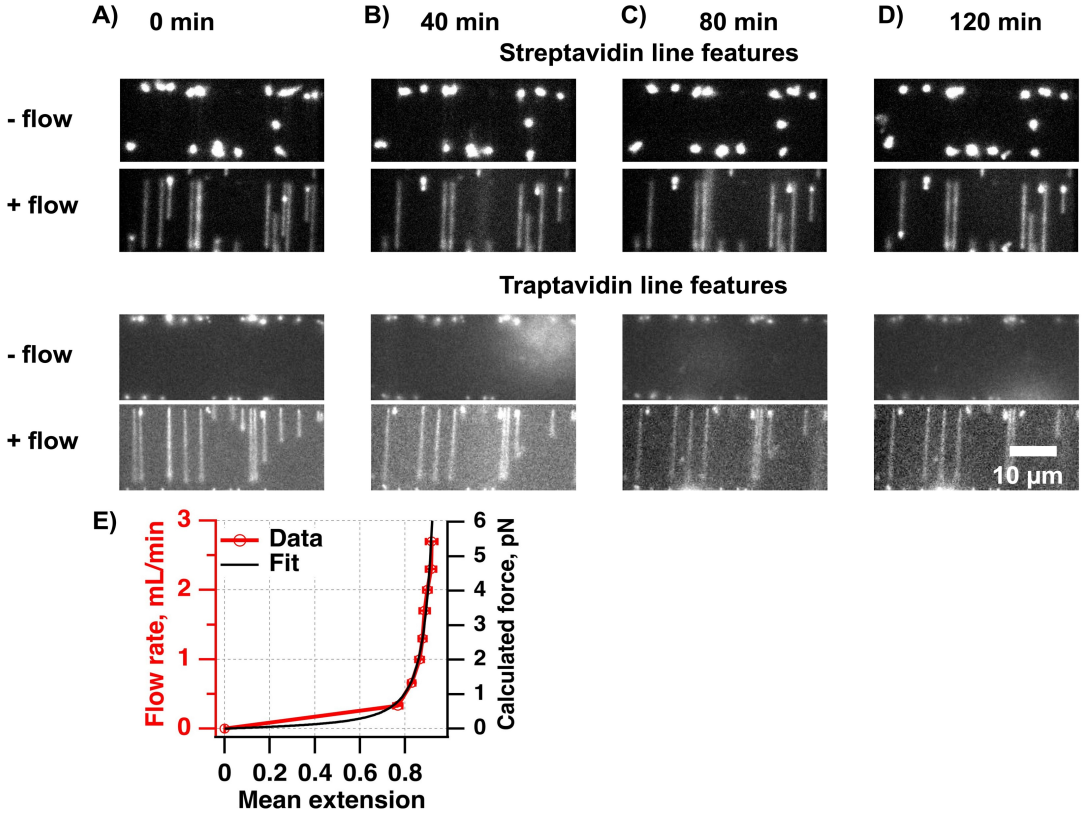

Figure S9: Stability test and shear flow extension of single-tethered Soft DNA Curtains. **A-D)** An example of single-tethered λ DNA molecules immobilized on a streptavidin (top panels) or traptavidin (bottom panels) array template, stained with SYTOX green (SG). Images were acquired every 20 min for a period of 2 h. In between acquisitions, there was no buffer flow applied. During the acquisition, 20 frames were acquired at buffer flow of 1 mL/min and 20 frames without the flow. Printing pressure was 0.6 mL, Si master #3, TIRF images were acquired at 488 nm wavelength excitation and averaged over 10 consecutive frames. **E)** Graph shows relative mean extension as a function of flow rate. Measured data points are shown in red with corresponding SD. Black line is representing a fit of the measured data to an equation describing the worm-like-chain model for DNA molecules. Modeling parameters: T = 295 K, persistence length of DNA ~50 nm.

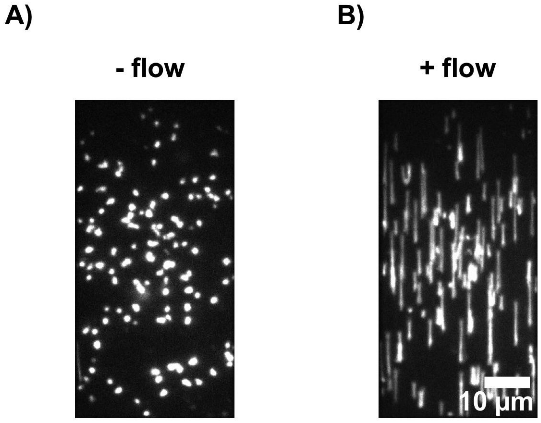

Figure S10: Functionality of surface immobilized biotinylated antibodies directed against digoxigenin (bt-anti-dig). The digoxigenated λ DNA molecules were immobilized on PEGylated (10% biotin-PEG) glass coverslip surface via bt-anti-dig and streptavidin (sAv, at 0.02 mg/mL). TIRF Images of SYTOX Green stained DNA molecules were acquired **A)** without or **B)** with buffer flow. These images were acquired at 488 nm wavelength excitation and averaged over 10 consecutive frames.

Table S3: Dependency of anchoring efficiency of the second DNA end on line-width of line-features.

Si master	Line-width of line-features (nm)	Separation distance between line-features (µm)	〈% of both-end anchored DNA〉 ± SD	〈# of both-end anchored DNA per line-feature〉 ± SD
#6	350	12	70.5 ± 34 (4.1 SEM, n=170)	1.6 ± 1.2 (0.1 SEM)
#4	500	12	73.9 ± 23.1 (2.9 SEM, n=307)	3.6 ± 1.8 (0.2 SEM)
#5	1000	12	78.6 ± 27.1 (3.5 SEM, n=380)	4.9 ± 3.2 (0.4 SEM)

Table S4: Dependency of anchoring efficiency of the second DNA end on line-separation distance of the tAv line-features.

Si master	Line-width of line-features (nm)	Separation distance between line-features (µm)	〈% of both-end anchored DNA〉 ± SD
#7	500	11	47.7 ± 28.8 (4.8 SEM, n=167)
#4	500	12	73.9 ± 23.1 (2.9 SEM, n=307)
#8	500	13	80 ± 16.7 (3 SEM, n=159)
#9	500	14	20.3 ± 22 (4 SEM, n=128)

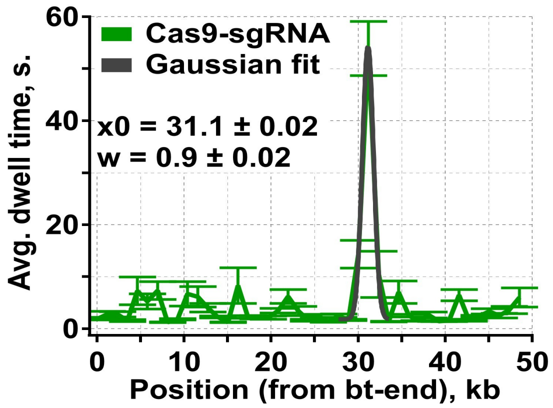

Figure S11: Histogram representing mean dwell time of Cas9-ATTO647N binding events. Data acquisition and the experimental scheme is explained in the Figure 3 of the main text.

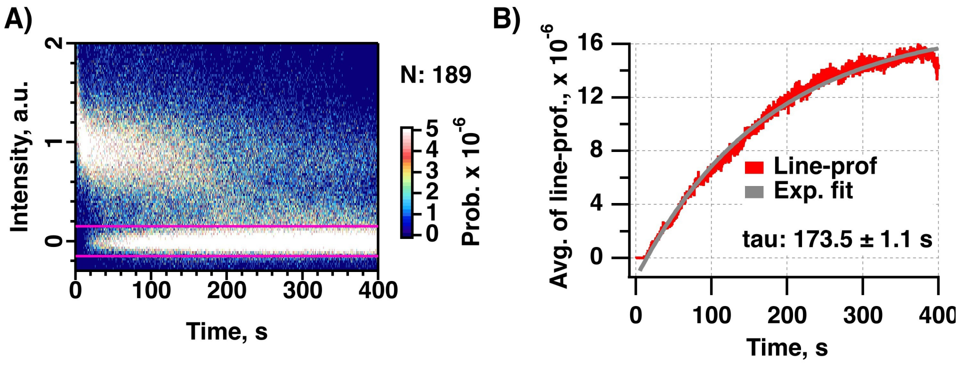

Figure S12: Photobleaching control of Cas9-ATTO647N immobilized on PLL coated glass coverslip surface. **A)** Population traces plot of overlaid Cas9-ATTO647N fluorescence emission traces acquired under constant illumination of 635 nm wavelength laser for 400 s. Traces were normalized to the beginning. **B)** Average horizontal line-profile taken on the population traces plot (panel A pink ROI) at the bleached region. The line-profile was fitted using single exponential function. The characteristic half-time for ATTO647N bleaching was ~ 173 s.

REFERENCES

(1) Elloumi-Hannachi, I.; Maeda, M.; Yamato, M.; Okano, T. Portable Microcontact Printing Device for Cell Culture. *Biomaterials* **2010**, *31* (34), 8974–8979. https://doi.org/10.1016/j.biomaterials.2010.08.019.

(2) Yaniger, S. I. Force Sensing Resistors™ a Review of the Technology. *Electro International, ELECTR 1991 - Conference Record*. 1991, pp 666–668. https://doi.org/10.1109/ELECTR.1991.718294.

(3) Gottipati S Lakshmi. Calibration of Force Sensitive Resistor Used in Force Controlled Grippers. *Int. J. Eng. Res.* **2020**, *V9* (08), 356–358. https://doi.org/10.17577/ijertv9is080129.
